# Mediastinal plasmacytoma with multiple myeloma presenting as a diagnostic dilemma

**DOI:** 10.1186/1757-1626-1-116

**Published:** 2008-08-21

**Authors:** Ashiq Masood, Kanan H Hudhud, AZ Hegazi, Gaffar Syed

**Affiliations:** 1 Cancer care center of Frederick, Frederick, MD, USA; 2 Frederick Medical Center, Frederick, MD, USA

## Abstract

Plasmacytoma are extramedullary accumulations of plasma cells. Most extramedullary **Plasmacytoma**s are associated with the upper respiratory tract. The mediastinum is rarely involved. We report a rare case of mediastinal plasmacytoma with multiple myeloma. The patient is 66 year old woman presented with bone pains and mediastinal mass on CT scan and MRI. The preliminary diagnosis of occult lung cancer with mediastinal involvement, and widespread skeletal metastasis was made, although lymphoproliferative disorder along with germ cell tumor was also kept in differentials.

The diagnosis of mediastinal plasmacytoma with multiple myeloma was made after extensive investigations

## Introduction

Plasmacytoma, a neoplastic proliferation of plasma cells, is a form of plasma cell dyscrasia that may manifest as multiple myeloma, primary amyloidosis, or monoclonal gammopathy of unknown significance. Plasmacytoma may be primary or secondary to disseminated multiple myeloma and may arise from osseous (medullary) or nonosseous (extramedullary) sites. Primary extramedullary plasmacytoma can be solitary or multiple [1]. The mediastinum is rarely involved by extramedullary plasmacytoma. We report a case of mediastinal plasmacytoma with multiple myeloma which is extremely rare in clinical practice. Our case highlights mediastinal plasmacytoma as differential diagnosis for mediastinal masses and aggressive search for multiple myeloma.

## Case presentation

**Figure 1:**
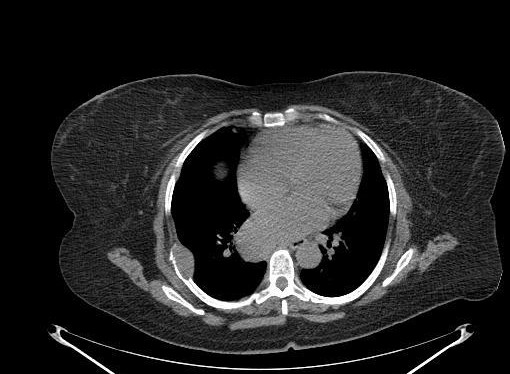
CT scan of chest showing mediastinal mass and pathological fracture of the right 8^th ^rib.

A 66 year old lady referred to our oncology clinic for the management of mediastinal mass. Two weeks prior to her visit, she started with severe low back pain radiating to lower extremities, nausea, retching, and mild dyspnea. She was admitted in the hospital, thorough investigation revealed mediastinal mass on CT scan (Figure 1). MRI of spine showed L4–L5 disc herniation along with significant spinal canal stenosis. She received treatment and was discharged in stable condition after 5 days of admission.

The patient's history was significant for GERD, Osteoporosis, and Hypercholestemia. She has been smoking less than a pack a day for 40 years and quit a month ago. Family history was remarkable for lung cancer (In her father, who died at the age 76) and sarcoidosis (in her only daughter). Current medications include rosuvastatin, omeprazole, Ibandronate, and ibuprofen.

On her first visit to our clinic, the patient was little uncomfortable due to bone pains, her blood pressure was 104/60 mmHg, heart rate of 84 beats per minute, respiratory rate 12 breathes per minute, and body temperature 99.6 F.

The cardiac and lung examination showed no murmurs, gallops, wheeze or rhonci. The rest of the examination which included HEENT, neck, abdomen, lymph nodes, and musculoskeletal was unremarkable.

**Figure 2:**
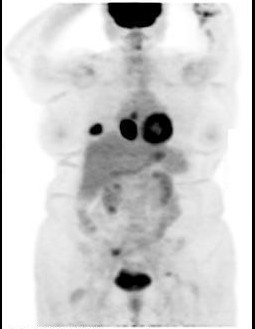
PET scan showing hypermetabolic activity in azygoesophageal recess and right 8^th ^rib.

The MRI showed 3.9 × 4.2 cms mass in azygoesophageal recess, the pathologic fracture of 8^th ^right rib, abnormal bone marrow signal in multiple areas of right sacroiliac joint, left superior acetabulum, and right greater trochanter. The PET scan confirmed these findings with uptake in all of these regions (Figure 2).

The provisional diagnosis of occult lung cancer with mediastinal involvement and wide spread skeletal metastasis was made, although the diagnosis of lymphoproliferative disorder and germ cell tumor was kept in differentials.

**Figure 3:**
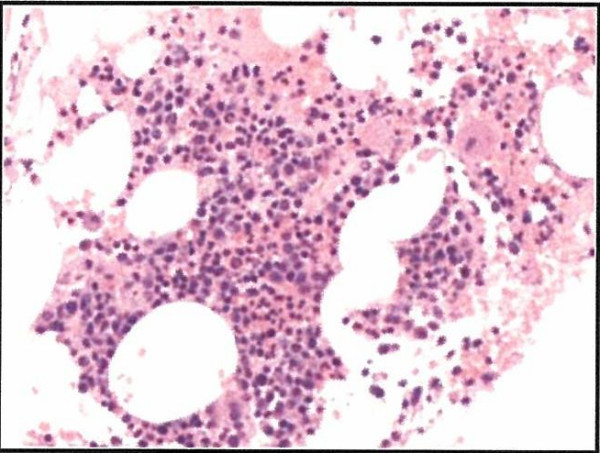
H&E stain illustrating trilineage hematopoiesis.

**Figure 4:**
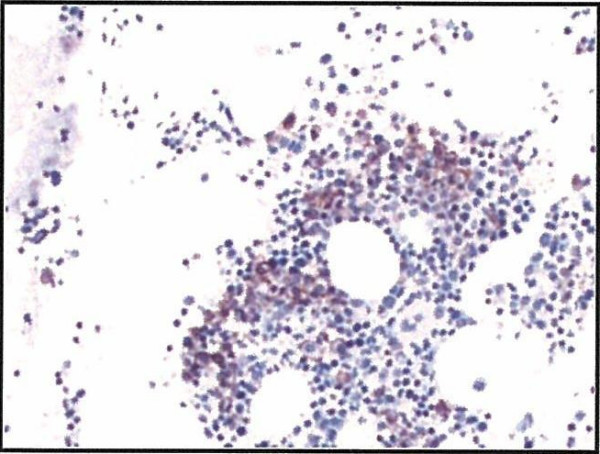
**CD138 stain demonstrating increased plasma cells**.

**Figure 5:**
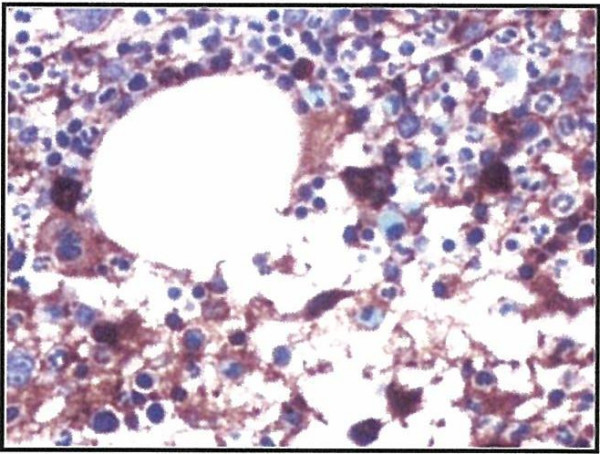
Plasma cells show excess lambda chain expression.

The patient underwent biopsy of mediastinal mass, which was found to be consistent with plasmacytoma (lambda light chain restricted). A bone marrow examination showed marrow involvement by plasma cell neoplasm (Overall 10% of the total cellularity). Figure 3, 4, and 5

Laboratory studies showed normochromic anemia (Hemoglobin level 107 g/l, MCV 86 fl), elevated ESR (61 mm/hr) and normal white cell and blood count. Beta microglobulin was elevated (5.2 mg/L) and BUN/Creatinine (21/1.3) was normal. Serum protein electrophoresis and immunofixation electrophoresis were negative but 24 hours urine for **protein **electrophoresis and immunofixation electrophoresis were consistent with free lambda chain measuring 85 mg/dl.

**Figure 6:**
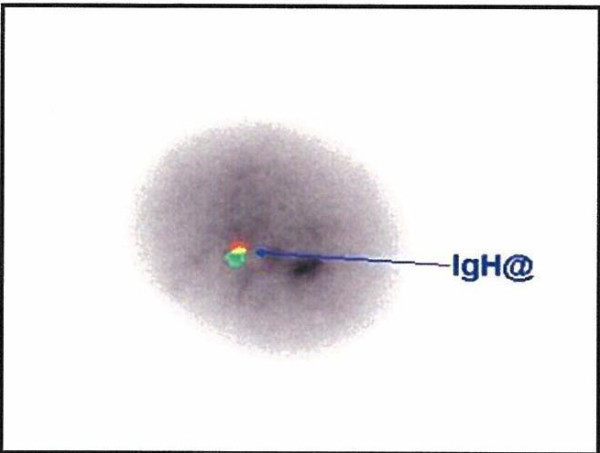
FISH showing abnormal result for chromosome 14 consistent with multiple myeloma.

Further workup included a cytogenetic/FISH analysis (showed abnormal result for chromosome 14, consistent with the presence of clonal lymphoid hematologic malignancy) Figure 6. Flow cytometry detected CD 56 + monoclonal plasma cells (1% of nucleated cells) also consistent with plasma cell disorder. The diagnosis of indolent multiple myeloma was made. The patient was put on chemotherapy with bortezomib and dexamethasone and is planned for autologous stem cell transplant (ASCT).

## Discussion

Extramedullary plasmacytoma (EMP) is a rare plasma cell neoplasm of the soft tissue and constitutes about 4% of all plasma cell tumors [2]. The most common site for extramedullary involvement is the upper aerodigestive tract [3]. The mediastinum is rarely involved by extramedullary plasmacytoma [3]. Only 5% of patients with EMP's have coexistent multiple myeloma [4]. However, in our case, a diagnosis of multiple myeloma was established within a month after the diagnosis of the mediastinal mass.

The sequences of proceedings suggest that the mediastinal plasmacytoma provided an early hint to the diagnosis of multiple myeloma, and therefore we conclude that the multiple myeloma was coexisting with the mediastinal lesion [5]. Our case is unique in the sense mediastinal plasmacytoma with multiple myeloma presenting simultaneously is extremely rare. The plasmacytoma in our case is aggressive revealed by the enhancer uptake on PET scan in distinction to the lack of increased uptake in indolent plasmacytoma [6,7]. Another aspect of our case is that it presented like a diagnostic dilemma; we initially thought that the diagnosis of occult lung cancer with mediastinal involvement with widespread skeletal metastasis. However, diagnosis of plasmacytoma with multiple myeloma was reached after extensive investigations.

After diagnosis of plasmacytoma aggressive search for multiple myeloma is vital as the management is entirely different for both types of Plasma Cell Dyscrasias

Plasmacytomas are treated with radiotherapy, surgery or both [8]. Chemotherapy may be considered for patients with refractory or relapsed disease [8] whereas multiple myeloma is mostly treated with chemotherapy [9]. Extranodal plasmacytoma in a patient with multiple myeloma carries a poor prognosis and treatment, which includes chemotherapy or autologous hematopoietic cell transplantation which is directed towards the underlying disease [10]. Our case also demonstrates the clinical usefulness of PET/CT scan in imaging plasmacytoma [6].

## Abbreviations

FISH: Fluorescent in situ hybridization; ASCT: Autologous stem cell transplantation.

## Patient consent

Written informed consent was obtained from the patient for publication of this case report and accompanying images. A copy of the written consent is available for review by the Editor-in-Chief of this journal.

## Competing interests

The authors declare that they have no competing interests.

## Authors' contributions

KHH conceived the study and provided substantial contributions to the analysis and interpretation of data. AM, the lead author involved in carrying out the literature search, study design and writing of case report. AZH and GS assisted with writing the paper. AZH also provided valuable insights in study design. KHH was involved in the diagnosis and management of case. All authors have read and approved the final manuscript.
